# Spinal Anesthesia

**Published:** 1901-08

**Authors:** 


### Abstracts and Selections.

### Spinal Anesthesia.

In the July 16th number of the *Philadelphia Medical Journal*, Dr. Angus McClain discusses the history and present status of spinal cocainization. The domain of cocaine as an analgesic is, he says, increasing. "First, it was used as a local external application on mucous membranes; second, a few minims of the stronger solutions were used subcutaneously to a limited area; third, it was used in diluted solution, combined with morphine and salt to extensive areas; fourth, it was injected into nerve trunks; fifth, it has been injected directly into the spinal canal, that it might have direct action on the lower portion of the spinal cord." As is customary with most surgeons, Dr. McClain uses one-fifth to one-third of a grain of cocaine in a two per cent. watery solution for injection into the sub-arachnoid space. The cocaine is best sterilized in dry envelopes and then dissolved in sterile water. Of the many needles in use perhaps the best is a finely drawn crucible needle with a trocar inserted, to be used with an all glass syringe. These can be sterilized by boiling and are so inexpensive that they can be thrown away after one using. The puncture is best made in the lamellar space between the fourth and fifth lumbar vertebra, though as good results may be obtained from going one or two vertebrae higher. The patient is required to sit with the spine flexed, and the spinous process of the fourth lumbar being used as a guide the needle is inserted laterally, about one-third of an inch from the spinous tip and directed inward and toward the median line until it enters the spinal canal. Some patients are completely anesthetized in from eight to twenty minutes, while others require one-half hour. The difference in susceptibility is explained by the larger amount of cerebro-spinal fluid in some subjects than in others, and by the position of the cocaine in the canal. Some patients become pale and nauseated while others suffer no ill effects. The most distressing after-effect is intense occipital headache, which may commence in from six or eight hours and last for three or four days. Dr. McClain has not succeeded in getting the good results from the use of tropococaine reported by W. Meyer and Schwartz. In this he agrees with Bier, who in addition reports unsatisfactory results from the use of eocaine.

The article concludes as follows: "As previously stated, I believe that lumbar puncture will have a useful position in the field of surgery, but I do not believe that the boundaries of its domain have yet been marked out. This cannot hope to supercede general anesthesia in all operations below the diaphragm until some method of technique or preparation of cocaine has been selected that will be followed by less distressing after-symptoms than we have from it as used at present.

